# Metabolic Characteristics and Response to High Altitude in *Phrynocephalus* *erythrurus* (Lacertilia: Agamidae), a Lizard Dwell at Altitudes Higher Than Any Other Living Lizards in the World

**DOI:** 10.1371/journal.pone.0071976

**Published:** 2013-08-07

**Authors:** Xiaolong Tang, Ying Xin, Huihui Wang, Weixin Li, Yang Zhang, Shiwei Liang, Jianzheng He, Ningbo Wang, Ming Ma, Qiang Chen

**Affiliations:** Institute of Biochemistry and Molecular Biology, School of Life Science, Lanzhou University, Lanzhou, China; University of Sao Paulo, Brazil

## Abstract

Metabolic response to high altitude remains poorly explored in reptiles. In the present study, the metabolic characteristics of *Phrynocephalus* *erythrurus* (Lacertilia: Agamidae), which inhabits high altitudes (4500 m) and *Phrynocephalus* *przewalskii* (Lacertilia: Agamidae), which inhabits low altitudes, were analysed to explore the metabolic regulatory strategies for lizards living at high-altitude environments. The results indicated that the mitochondrial respiratory rates of *P* *. erythrurus* were significantly lower than those of *P* *. przewalskii* , and that proton leak accounts for 74~79% of state 4 and 7~8% of state3 in *P* *. erythrurus* vs. 43~48% of state 4 and 24~26% of state3 in *P* *. przewalskii* . Lactate dehydrogenase (LDH) activity in *P* *. erythrurus* was lower than in *P* *. przewalskii* , indicating that at high altitude the former does not, relatively, have a greater reliance on anaerobic metabolism. A higher activity related to β-hydroxyacyl coenzyme A dehydrogenase (HOAD) and the HOAD/citrate synthase (CS) ratio suggested there was a possible higher utilization of fat in *P* *. erythrurus* . The lower expression of PGC-1α and PPAR-γ in *P* *. erythrurus* suggested their expression was not influenced by cold and low PO_2_ at high altitude. These distinct characteristics of *P* *. erythrurus* are considered to be necessary strategies in metabolic regulation for living at high altitude and may effectively compensate for the negative influence of cold and low PO_2_.

## Introduction

High altitude is a major challenge to life; however, native people and animals can survive vigorously in the cold and hypoxic conditions associated with these environments [1,2]. These endemics are adapted to high altitudes in various ways, including morphological traits, haematological characteristics, thermogenesis and metabolism, etc [3–5]. Such adaptations are conducive to organisms being able to maximize their fitness and alter the reaction norms of phenotypes in response to various biotic and abiotic factors, thus give them a “head start” in dealing with environmental changes [6]. However, compared with endothermic animals, ectothermic animals living in high altitude habitats have to manage certain additional physiological challenges, including fluctuating body temperature and sustaining metabolic rates in conditions of reduced oxygen availability [1]. Consequently, a highly efficient and well-regulated metabolism to counter the impacts of extreme environmental conditions is necessary and important for endemic ectothermic animals at high altitude.

Over recent decades, in order to investigate the characteristics and adjustment ability of metabolism in response to temperature fluctuation and oxygen concentrations in ectothermic vertebrates, many studies have been conducted on the effects of these two factors on the mitochondrial respiratory rate, metabolic enzyme activity and metabolism-related genes [7–9]. As the site of aerobic metabolism, mitochondria form a ‘hot topic’ in the study of metabolic characteristics and mechanisms. Mitochondrial respiratory rate strongly reflects metabolic adjustments in cells [10]. Some intraspecific comparisons between populations from different altitudes have revealed notable variations in the mitochondrial metabolism and have predicted that such variations could be induced by cold temperature and low partial pressure of oxygen (PO_2_) at high altitude [11,12]. Proton leak or proton cycling is another key regulator in the utilization of oxygen in the mitochondria, which partially uncouples oxygen consumption from adenosine triphosphate (ATP) synthesis, leading to less effective energy conservation [13]. Both the capacity of mitochondrial oxidation and proton leak may be affected by the fatty acid composition of the mitochondrial inner membrane and membrane potential, which may vary throughout an animal’s life history according to changes in body mass or age, as well as in response to environmental conditions such as temperature and oxygen levels [5,14]. In addition, metabolic enzyme activity may be altered in response to different environmental conditions by changing rates of transcription or by expressing allozymes and isozymes according to different thermal sensitivities [15]. Adjustment of enzyme activities may be of particular importance to reptiles which inhabit at high altitudes. Not only are these animals continuously subjected to low partial pressures of oxygen but they also have the added metabolic cost while being exposed to extremely low ambient temperatures. Studies on hibernating ectothermic vertebrates in conditions of low temperature and low PO_2_ similar to a high altitude environment reveal a significantly decreased enzyme activity that may be induced by a combination of low temperatures and anoxic conditions [16,17].

So far, studies on the mechanisms of high altitude adaptation in reptilian species have mainly been conducted using acute or chronic treatments (from several days to months) of low temperature and/or low PO_2_ [18,19]. These treatments generally only induce adjustments at the level of mitochondria and enzymes; however, the adaptation characteristics of native reptilian species at high altitude are considered to be the result of long-term evolution at high altitude and may be strongly heritable. So, in this way, adaptation of native animals at high altitude mainly occurs at the genetic level, and may be accompanied by a new integration of cell metabolism [11]. Reported data suggest that certain genes should be related to endothermic vertebrates metabolic regulation, and much information has been gathered on the effect of cold temperature and low PO_2_ on the expression of genes, such as PGC-1α (peroxisome proliferator-activated receptor γ coactivator-1) and PPAR (peroxisome proliferator-activated receptors). PGC-1α is the best-studied coactivator and plays a central role in the regulation of cellular energy metabolism [20]. PPAR activity is regulated by PGC-1, originally identified as a transcriptional coactivator of the nuclear receptor PPAR-γ, which regulates the activity of several nuclear receptors. Many hypotheses about the physiological role of these two genes in mammals have been formed, including the regulation of energy metabolism or control of body mass. However, its functions remain unclear in reptiles. The expression level of these genes may significantly vary among species, and its regulation could be complicated by simultaneous changes in cold temperature and low PO_2_ conditions [21,22]. There remains an inadequate understanding of the functions of these genes in reptiles and, to our knowledge, no information about these genes has yet been gathered for lizards endemic to high altitudes.

The influence of cold and low PO_2_ on the metabolism of lizards living at high altitude remains relatively unexplored, since lizards are not abundantly distributed at altitudes above 4000 m [23–26]. However, some viviparous toad-headed lizards are widely distributed on the Tibetan plateau from altitudes of 2500 m to 4800 m [27]. The red tail toad-headed lizard *Phrynocephalus* *erythrurus* (Lacertilia: Agamidae), which lives on the Qiangtang Plateau (mostly 4500–5300m above sea level) in northern Tibet, is considered to be the highest living lizard in the world [27]. This species, initially described by Zugmayer (1909), is one of the least studied reptile species in China; we were unable to find any published work on this species, other than for its phylogeography [28]. Another species, *Phrynocephalus* *przewalskii* (Lacertilia: Agamidae), which inhabits desert and semi-desert areas in north China (altitude from 1000 to 1500m), was selected as a reference species for the present study. The environmental conditions of the habitats of these two lizards are very different, and the high altitude *P* *. erythrurus* has to manage long-term extreme cold temperatures and low PO_2_ (Table 1). Here, these two closely-related species are used to analyse differences in the mitochondrial respiratory rate, some metabolic enzymes and metabolism-related genes in both the liver and skeletal muscle, in order to accumulate some useful information on lizard metabolic characteristics and provide new insights into the adaptation mechanisms of reptiles at high altitude.

**Table 1:** Climatic data of Tuotuo River and Minqin from 1959 to 2009.

Location	Tuotuo River					Minqin				
Meteorological parameter	Mean temperature (°C)	Highest mean temperature (°C)	Lowest mean temperature (°C)	Atmospheric pressure (hPa)	Sunshine duration (Hour)	Mean temperature (°C)	Highest mean temperature (°C)	Lowest mean temperature (°C)	Atmospheric pressure (hPa)	Sunshine duration (Hour)
Annual Values	-3.99	4.42	-11.04	587.79	7.97	8.29	16.08	1.34	863.73	8.45
April	-3.69	5.22	-11.85	587.41	8.41	10.53	18.52	2.84	862.07	8.67
May	1.12	9.17	-6.07	585.55	8.83	16.82	24.38	8.86	860.49	9.44
June	5.14	12.32	-0.90	589.99	8.32	21.29	28.79	13.34	857.65	9.84
July	7.74	14.65	2.05	590.88	8.15	23.36	30.71	16.12	856.27	9.33
Augest	7.28	14.16	1.67	591.95	8.09	21.78	29.06	15.05	858.95	9.09
September	3.73	10.92	-1.65	591.96	7.93	15.98	23.58	9.33	863.80	8.38
October	-3.85	4.39	-10.04	591.11	8.58	8.17	16.46	1.38	867.96	8.17

## Materials and Methods

All experiments were carried out according to protocols approved by the Ethics Committee of Animal Experiments at Lanzhou University and in accordance with guidelines from the China Council on Animal Care. Both liver and skeletal muscle were harvested by surgery. All surgery was performed under sodium pentobarbital anaesthesia, and every effort was made to minimize the numbers used and any suffering experienced by the animals in the experiments.

### Animal collection and maintenance

The *P* *. erythrurus* and *P* *. przewalskii* individuals were captured from the Hoh-xil National Nature Reserve and Minqin Integrated Desert Control Experiment Station, respectively. Both areas are protected and only used for scientific research, and the two authorities gave us permission to capture the animals used in this study. Meanwhile, our field studies did not involve endangered or protected species. The periods of the two lizards’ collection were from June to July 2012. The lizards were active in their habitat during this season. The mating have already completed in both *P* *. erythrurus* and *P* *. przewalskii* , and only male lizards were used in our present study, so we could exclude the influence of reproduction on their metabolism. Toad-headed lizards *P* *. erythrurus* were captured by hand in the wild at Tuotuo River (34°13'N, 92°13'E), Qinghai province, China, in August 2012 (N=35). The mean body mass was 5.26±0.21 g and mean snout-vent length (SVL) was 4.98±0.32 cm. *P* *. przewalskii* individuals were collected from Minqin (38°38'N, 103°05'E), Gansu province, China, in July 2012 (N=42). The mean body mass and SVL were 5.75±0.38 g and 5.61±0.44 cm, respectively. The elevations of the two collection sites (measured by GPS, Magellan Explorist 600) were 4543 m and 1482 m, respectively. Climatic data (atmospheric pressure, highest and lowest temperature, air temperature and mean sunshine duration) of the two sampling sites were provided by the Chinese Climatic Data Centre (CDC) for the years from 1959 to 2009. All these climatic data were recorded at meteorological stations, which were situated at or near (< 2 km) the collection sites.

All captured lizards from the collecting zone were brought to the laboratory at Lanzhou University (36°05'N, 103°86'E) within 24 hours of capture. These lizards were maintained in a room at constant temperature (16±0.5°C), which was controlled by an air-conditioning system. As the habitat environmental conditions of *P* *. erythrurus* are quite different from our laboratory environment, especially the difference in PO_2_, we needed to limit the possible effect of changed PO_2_ on the physiological and biochemical characteristics of the lizards. *P* *. erythrurus* individuals were therefore kept in a non-pressurized hypoxic chamber (100 cm length, 45 cm width and 45 cm height) to simulate a low PO_2_ at an altitude of 4550 m. Nitrogen gas was continuously added to the chamber to dilute the oxygen concentration to 11.8% (PO_2_^≈^90 mmHg, equivalent altitude of 4550 m). After the required oxygen concentration reached, it was then monitored and maintained by an oxygen controller (HCD-2B, Mei Cheng Oxygen Analysis Instruments Plant) connected to an electromagnetic valve to control the nitrogen ﬂow. A 60 W bulb was suspended above one end of the chamber and operated for 10 h (from 0830h to 1830h) to provide a thermal gradient from 35 to 16°C. The bottom of the chamber was covered with a 10-mm depth of silver sand, and several bricks were placed in the chamber to give the lizards the opportunity to choose their optimal temperature or basking place. Meanwhile, fluorescent lamps were used to simulate natural light on a constant 12h: 12h light on: off cycle for each chamber. The climatic conditions and altitude of Lanzhou is similar to the Minqin, so the *P* *. przewalskii* were simply maintained in the chamber with PO_2_=137mmHg. The other conditions were the same as for *P* *. erythrurus* . All lizards were fed mealworms and water *ad libitum* and all experiments were finished within 10 days of the collection.

### Tissue sampling

The liver and skeletal muscle were blotted with absorbent paper to remove excess liquid and then weighed (accuracy: 0.001 g). The fresh tissues were used for mitochondrial assay, and the remaining tissues were immediately frozen in liquid nitrogen and then transferred into a cryogenic refrigerator and frozen at −80° C for subsequent assays of enzyme activity and gene expression.

### Mitochondrial isolation

For both species, 10 adult male lizards were used in the mitochondrial assay. The mitochondrial isolation was conducted according to published protocols with the following modifications. For liver mitochondria, the fresh organ was weighed and immediately homogenized in four volumes of ice-cold isolation buffer (250 mmol l^-1^ sucrose, 5 mmol l^-1^ Tris/HCl, pH 7.4, and 2 mmol l^-1^ EGTA). The homogenate was spun at 3000 rpm for 5 min at 4° C, and the supernatant centrifuged at 12000 rpm for 10 min at 4° C. Then, the supernatant was discarded and the mitochondria were resuspended in an 800-µl isolation medium. In order to isolate the mitochondria of the skeletal muscle, the gastrocnemius from the hind-leg was removed, weighed and cut with scissors and homogenized in the isolation buffer (140 mmol l^-1^ KCl, 50 mmol l^-1^ Tris/HCl, pH 7.4, 2 mmol l^-1^ EGTA, 0.5% (w/v) BSA, 5 mmol l^-1^ MgCl_2_, 1 mmol l^-1^ ATP and 2.45 units ml^-1^ Protease Type VIII), using a glass homogenizer. The homogenate was centrifuged at 3000 rpm for 5 min at 4° C, and the resulting supernatant was subjected to a high-speed spin cycle (12000 rpm, 10 min and 4° C). The mitochondrial pellet was resuspended in 650 µl medium (140 mmol l^-1^KCl, 50 mmol l^-1^ Tris/HCl, pH 7.4, 2 mmol l^-1^ EGTA, 0.5% (w/v) BSA).

### Mitochondrial O_2_ consumption and proton leak measurement

Mitochondrial respiration was monitored on a Chlorolab 2 system (Hansatech Instruments, Norfolk, England) and thermostatically controlled at constant temperatures (20 and 30°C) by a circulator bath. The resulting mitochondrial solution (140 µl pre-assay) was saturated by room air with steadfast stirring at 50 rpm. The respiratory rate of state3 was determined in the presence of 5 mmol l^-1^ succinate and 5 µmol l^-1^of rotenone (to inhibit complex I of the respiratory chain) after the addition of 1 mmol l^-1^ ADP, and the respiratory rate of state4 was measured after all the ADP was consumed. Thermal sensitivities were expressed as Q_10_ = (K2/K1)^10 /T2–T1^, where K1 and K2 are the reaction rates at temperatures T1 and T2, respectively.

Evaluation of possible uncoupling mechanisms followed the methods given by Guderley et al. and Rey et al., with some modifications [29,30]. Liver and skeletal muscle mitochondria from the fresh organs were used and the assays proceeded at 30° C. The potential contribution of UCP to mitochondrial respiration was evaluated by measuring inhibition of state4 using the addition of 2 mmol l^-1^ guanosine diphosphate (GDP). The impact of inhibiting the adenine nucleotide translocase (ANT) was measured in an independent set of experiments and assessed by the addition of a non-competitive inhibitor, carboxyatractyloside (CAT), to a final concentration of 5 µM.

### Enzyme activity measurement

A total of 10 lizards from each species were used for the analyses of enzyme activity. Tissues (liver and skeletal muscle) for lactate dehydrogenase (LDH, EC 1.1.1.27), citrate synthase (CS, E.C. 2.3.3.1) and β-hydroxyacyl coenzyme A dehydrogenase (HOAD, EC 1.1.1.35) assays were collected after the lizards were brought to our laboratory and stored at −80° C until analysis. Our methods for LDH and CS measurements followed those of Seebacher et al. [31], and the HOAD assay was conducted according to the published protocols of John-Alder and Joos [32], with some modifications. Tissue samples (0.05–0.15 g) were homogenized in nine volumes of ice-cold extraction medium (100 mmol l^-1^ potassium phosphate (KH_2_PO_4_/K_2_PO_4_), 2 mmol l^-1^ MgCl_2_, 5 mmol l^-1^EDTA, 1 mmol l^-1^ reduced glutathione and 1% Triton X-100), and enzyme activities were determined in a temperature-controlled spectrophotometer (UV2000, Unico Instrument Co., Ltd., Shanghai, China). All assays were performed in duplicate at 20 and 30°C and enzyme activity was expressed in units of g^-1^ wet tissue.

### RNA isolation and cDNA synthesis

Total RNA was extracted from 50–100mg of liver and muscle using RNAiso Plus reagent (Takara Bio, Japan) according to the manufacturer’s instructions. RNA concentration and purity was measured using the NanoDrop 2000 (Thermo Scientific, USA) and the integrity was confirmed using 1.2% agarose gel (Gene Tech, Shanghai, China). The residue of genomic DNA was removed in a volume of 10 µl at 42° C for 2 min, containing 1 µg of RNA samples, 2 µL 5×gDNA Eraser Buffer, 1 µL gDNA Eraser and RNase Free dH_2_O. Immediately after this, first-strand cDNA synthesis was performed by adding 4 µL of 5× PrimeScript Buffer, 1 µL of PrimerScript RT Enzyme Mix, 1 µL of RT Enzyme and 4 µL RNase Free dH_2_O at 37 °C for 15 min, 85 °C for 15 s.

### RT-PCR ampliﬁcation and quantitative real-time PCR

We designed new primers for the partial sequence of PGC-1α and PPAR-γaccording to the sequences of *Anolis* *carolinensis* and *Eublepharis* *macularius* obtained from GenBank; all primer sequences are described in Table 2. The polymerase chain reaction (PCR) amplification was conducted in a total volume of 20 µL containing 10×PCR buffer, 3 mM Mg^2+^, 0.50 µM of each primer, 0.50 mM of dNTPs, 0.50 units of Taq DNA polymerase (Sangon, Shanghai, China) and about 100 ng cDNA templates. Touchdown PCR protocols were used: 95° C for 5 min, 20 cycles of 95° C for 30 s, 65° C for 30 s, reducing by 2° C every two cycles to 51° C and 72° C for 1 min; 15 cycles at 55° C and 72° C for 10 min. PCR products were visualized on 2% low melting agarose gels. Bands were cut, purified with an EZ spin column DNA gel extraction kit (Sangon, China) and cloned into PMD18-T Vector (Takara Bio, Japan), and subsequently sequenced.

**Table 2:** Primer sequence used for RT-PCR ampliﬁcation.

Primers name	Primer sequence(5’- 3’)	Product length/bp
PGC-1α-F	TCAAGGTCACCATGCAGTAGAT	518
PGC-1α-R	CTTCGCTGTGCCTCTTTAAGTA	
PPARγ-F	ATTAGCCCAGTGGACCTTTCT	676
PPARγ-R	TTGTCTTTCCCGTCAAGATTG	
ACTB-F	TGATGGTGGGCATGGGNCARAARGA	290
ACTB-R	CACGGCCTGGATGGCNRCRTACAT	

Expression patterns of these target genes were analysed using quantitative real-time PCR and β-actin was selected as an internal control for gene expression. We designed the speciﬁc primers for target or reference genes using the Primer Premier 5 (Table 3). The amplifications were performed in a 96-well plate in a 20 µL reaction volume containing 10 µl of 2×SYBR Master Mix Takara Bio, Japan), 0.4 µM of each primer, 2 µL of cDNA template, and DEPC-water. The PCR cycles were 95° C for 30s, 39 cycles of 95° C for 5s and Tm for 30s. All assays were performed in triplicate on 96-well plates, and for each of these we also conducted negative (RNA samples) and blank (water) controls using an CFX-96 real-time PCR machine (Bio-Rad Laboratories, Inc., CA, USA). The relative expression was calculated as 2^–ΔΔCt^ [33].

**Table 3:** Primer designed for gene expression analysis.

Primers name	Primer sequence(5’ -3’)	Product length/bp	Tm
PGC-1α-Q-F	AAAACGGGAATCTGAAAGGG	134	55.0
PGC-1α-Q-R	ATACTTCAAACCGGTCTGTC		
PPARγ-Q-F	CTCCCATGCCTTCGACATAA	139	53.0
PPARγ-Q-R	GCATTCTTGGAACTTCACAT		
ACTB-Q-F	CCCATTGAGCACGGCATT	146	57.0
ACTB-Q-R	CTTTTCCCTGTTGGCTTTGG		

### Statistical analyses

All data were tested for normality and homogeneity of variances to meet the assumptions of parametric testing prior to analysis, and no significant deviations from these assumptions were evident in the data. Data on morphological traits, values of Q_10_ and gene expression were analysed using an ANOVA followed by a post-hoc Tukey’s test, and means values of mitochondrial uncoupling were compared using a paired t-test. Potential interactions between activity states (mitochondria and enzymes) and assay temperature were analyzed by two-way ANOVA. The values were reported as Means ± Standard error (s.e.m.) and were performed using SPSS release 16.0.0 (SPSS, Inc., Chicago, Illinois, USA).

## Results

### Respiratory rate of liver and skeletal muscle mitochondria

**Figure 1:**
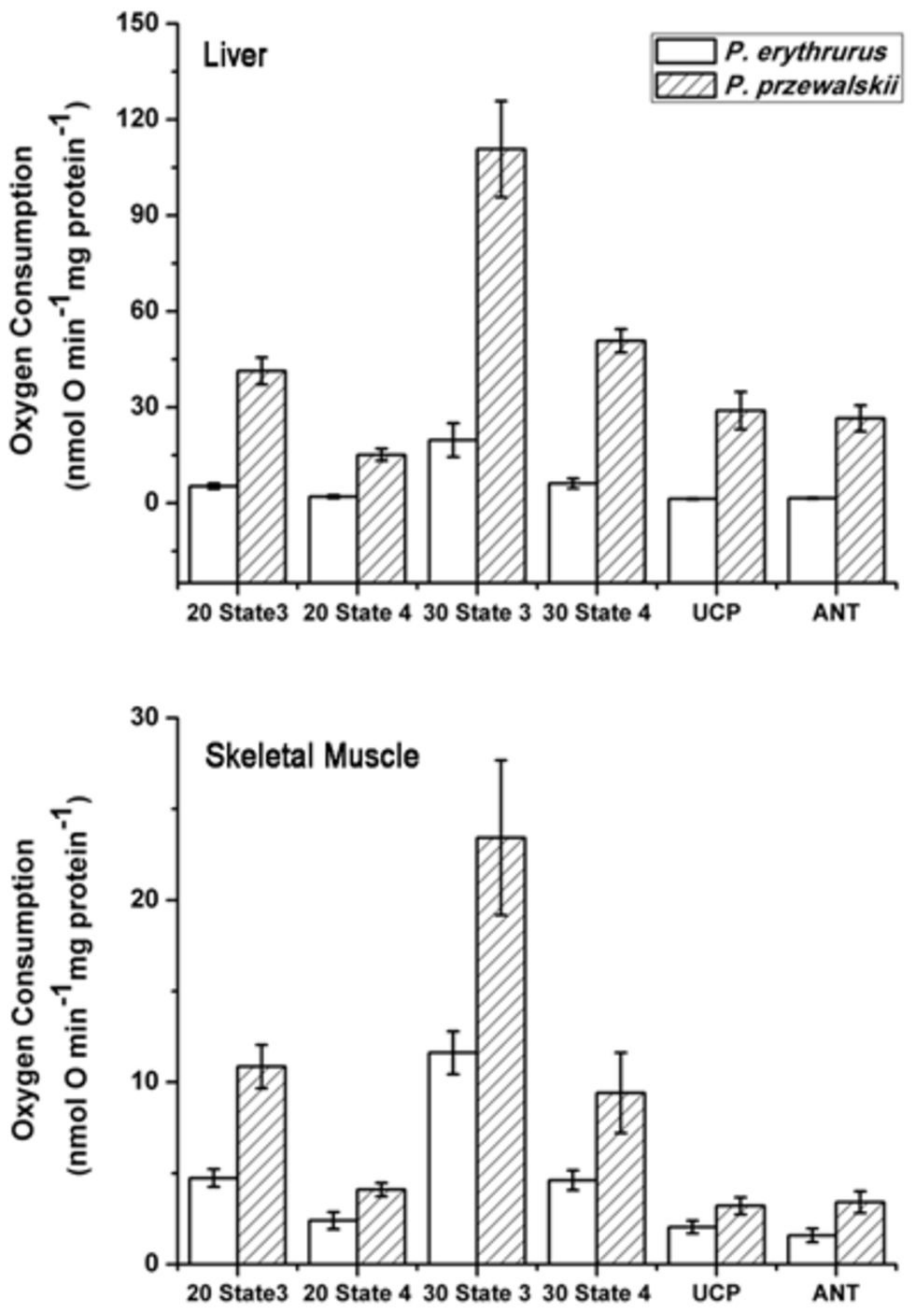
Mitochondrial respiratory rate and the uncoupling of liver and skeletal muscle in *Phrynocephalus* *erythrurus* and *Phrynocephalus* *przewalskii* , respectively. Values are means ± s.e.m.

For both liver and skeletal muscle mitochondria, the respiration rates of state3 and state4 increased with assay temperatures in the two species (Figure 1). Although the mean Q_10_ was not significantly different between the two species, the values of Q_10_ for state 3 and state 4 of both the liver and skeletal muscle in *P* *. erythrurus* were higher than those in *P* *. przewalskii* . The only exception was the mean Q_10_ value of state 4 in *P* *. erythrurus* , which was lower than that in *P* *. przewalskii* (Table 4).

**Table 4:** Thermal sensitivity (Q_10_) of mitochondrial oxidation and enzyme activities in different tissues of *Phrynocephalus* *przewalskii* and *Phrynocephalus* *erythrurus* .

		*Phrynocephalus* *przewalskii*	*Phrynocephalus* *erythrurus*	Interspecific effect F
Liver	State3	2.75±0.24	4.02±0.65	2.072 ns
	State4	3.87±0.24	3.17±0.43	1.716 ns
	LDH	2.23±0.16	2.48±0.35	0.434 ns
	CS	2.41±0.34	2.86±0.42	0.670 ns
	HOAD	1.70±0.40	1.16±0.17	1.906 ns
Skeletal muscle	State3	1.38±0.57	2.69±0.44	3.434 ns
	State4	1.65±0.57	2.89±0.71	1.511 ns
	LDH	2.05±0.08	2.07±0.04	0.104 ns
	CS	5.24±2.08	3.89±1.34	0.244 ns
	HOAD	2.81±0.72	1.94±0.14	1.930 ns

We also compared the possible interspecies variations in state3 and state4 for liver and skeletal muscle mitochondria, respectively. For liver mitochondria, the rate of state3 in *P* *. erythrurus* was much lower than that in *P* *. przewalskii* at the two assay temperatures (20° C:F_1, 11_ = 43.358, P<0.001; 30° C:F_1, 12_ = 25.250, P<0.001). Rates of state4 were also lower in *P* *. erythrurus* than in *P* *. przewalskii* at both 20 (F_1,11_=29.940, P<0.001) and 30° C (F_1,13_=113.065, P<0.001). At both assay temperatures, the maximal rates of liver mitochondria in *P* *. przewalskii* were approximately five- to eight-fold higher than in *P* *. erythrurus* ; similarly, the oligomycin-inhibited rates of *P* *. przewalskii* were almost seven- to eight-fold higher than for *P* *. erythrurus* (Figure 1). On the other hand, skeletal muscle mitochondria in *P* *. przewalskii* had significantly higher maximal rates of succinate oxidation than the mitochondria of *P* *. erythrurus* at both assay temperatures (20° C:F_1, 9_ = 26.042, P<0.001; 30° C:F_1, 14_ = 8.963, P=0.010). There were also significant differences in the rate of state4 between the two species; the oligomycin-inhibited mitochondria from *P* *. erythrurus* had a lower respiratory rate than those from *P* *. przewalskii* . The rates of state3 and state4 at both assay temperatures were approximately twice as fast in *P* *. przewalskii* as in *P* *. erythrurus* (Figure 1).

### Mechanisms of mitochondrial uncoupling

In liver, GDP significantly reduced the oxygen uptake of oligomycin-inhibiting state4 respiration in *P* *. przewalskii* (N=6, paired t-test, t=7.211, P=0.001) and *P* *. erythrurus* (N=5, paired t-test, t=4.311, P=0.013), respectively. However, the addition of CAT reduced oxygen uptake, but only in *P* *. przewalskii* (N=7, paired t-test, t=4.103, P=0.006), not in *P* *. erythrurus* (N=6, paired t-test, t=2.447, P=0.058). For skeletal muscle mitochondria, neither GDP (N=4, paired t-test, t=2.390, P=0.097) nor CAT (N=4, paired t-test, t=2.593, P=0.122) significantly reduced the oxygen uptake in *P* *. przewalskii* . On the other hand, the reverse results were found in the skeletal muscle mitochondria of *P* *. erythrurus* ; both GDP (N=5, paired t-test, t=5.434, P=0.006) and CAT (N=5, paired t-test, t=3.895, P=0.018) dramatically reduced oxygen uptake at 30° C (Figure 1).

We also analysed possible interspecies differences related to the effect of GDP and CAT on the oxygen uptake of liver or skeletal muscle mitochondria (Figure 1). In liver, *P* *. przewalskii* mitochondria were inhibited by GDP (F_1,15_=8.879, P=0.009) and CAT (F_1,17_=16.765, P=0.001), and had a significantly higher respiratory rate than those from *P* *. erythrurus* . Similar to the results for liver mitochondria, CAT-inhibited skeletal muscle mitochondria from *P* *. przewalskii* consumed oxygen faster than those from *P* *. erythrurus* (F_1,11_=5.110, P=0.045). However, inhibition by GDP did not significantly affect the respiratory rate between the two species (F_1,11_=3.758, P=0.079).

### Metabolic enzyme activity

**Figure 2:**
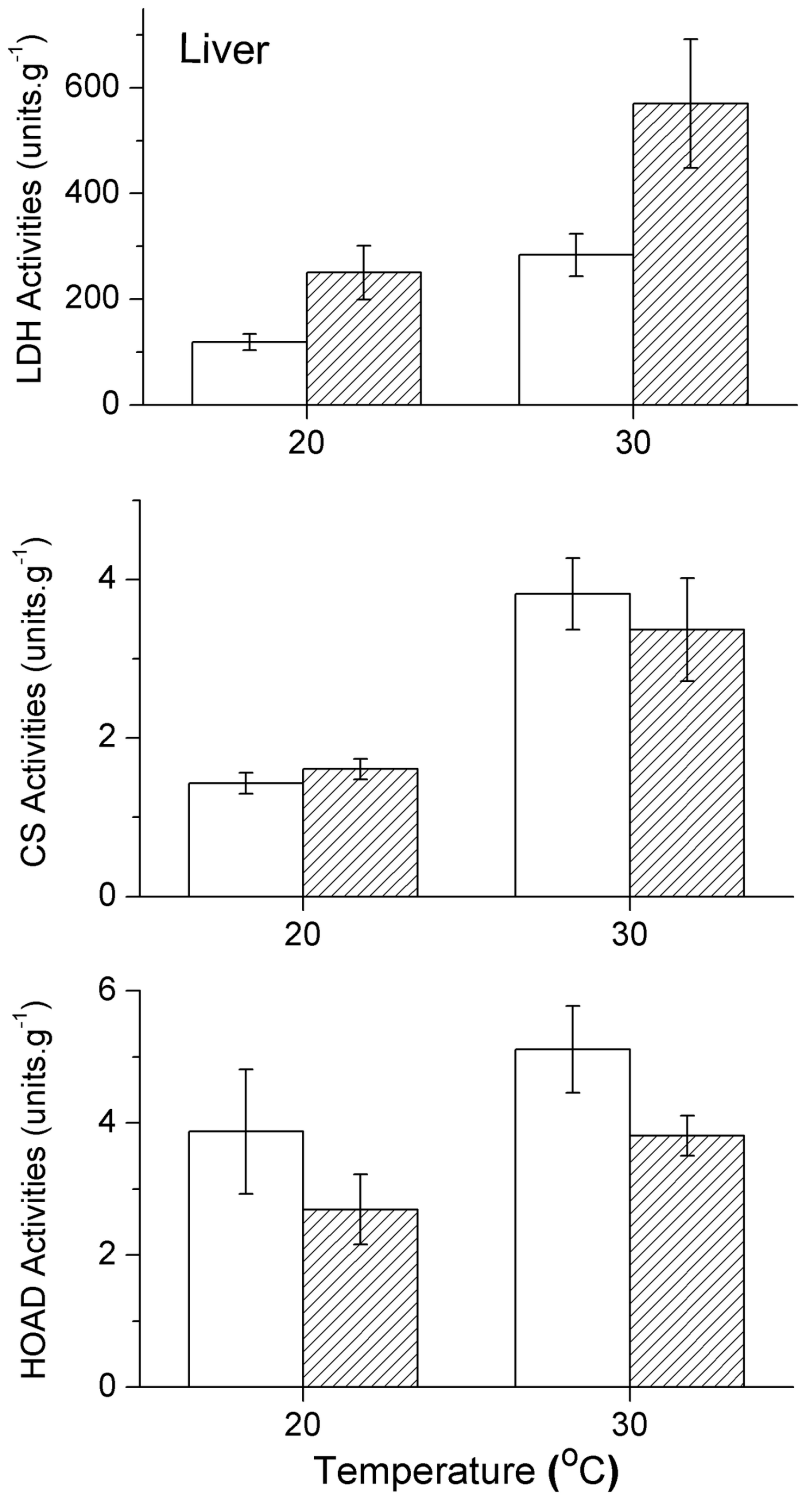
Activities of lactate dehydrogenase (LDH), citrate synthase (CS) and β-hydroxyacyl coenzyme A dehydrogenase (HOAD) at 20 and 30°C in the liver of *Phrynocephalus* *erythrurus* (Open columns) and *Phrynocephalus* *przewalskii* (Twill columns). Values are means ± s.e.m.

**Figure 3:**
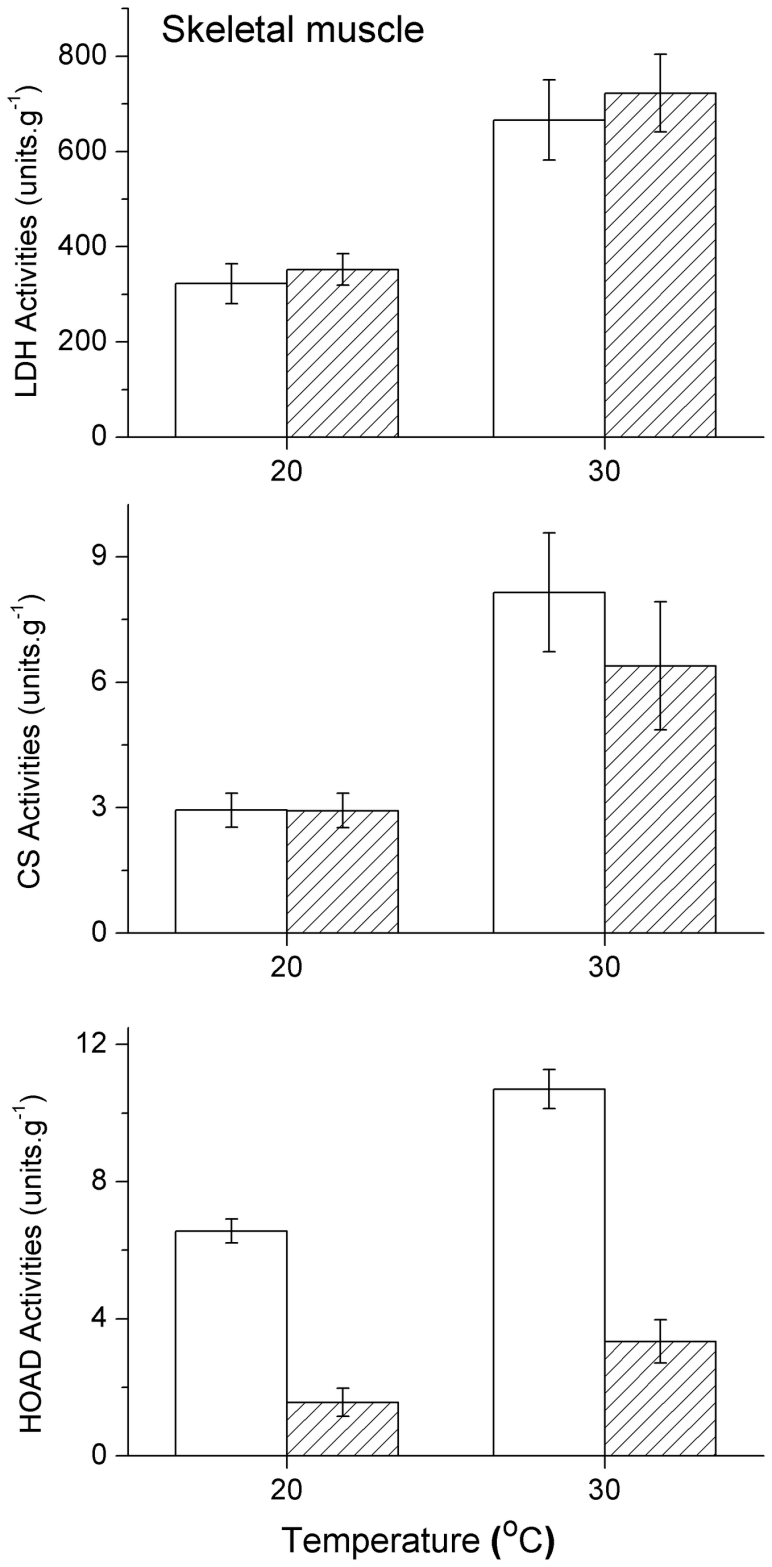
Activities of lactate dehydrogenase (LDH), citrate synthase (CS) and β-hydroxyacyl coenzyme A dehydrogenase (HOAD) at 20 and 30°C in the skeletal muscle of *Phrynocephalus* *erythrurus* (Open columns) and *Phrynocephalus* *przewalskii* (Twill columns). Values are means ± s.e.m.

In both the liver and skeletal muscle of the two species, LDH activity increased significantly with the assay temperatures (all F>5.982, P<0.026). The differences in LDH activity between the liver and skeletal muscle of the two species were also analysed. Liver LDH activity in *P* *. przewalskii* was notably higher than in *P* *. erythrurus* at 20° C (F_1,18_=6.092, P=0.024) and 30° C (F_1,18_=4.999, P=0.038) (Figure 2), but no significant difference was observed in the skeletal muscle of either of the two species at both assay temperatures (20° C:F_1, 17_ = 0.300, P=0.591; 30° C:F_1, 17_ = 0.230, P=0.638) (Figure 3).

The assay temperatures had a significant effect on the CS activity in all tissues and both species (all F>4.743, P<0.050). However, there was no significant difference in CS activity for either liver or skeletal muscle between the two species at 20 and 30°C, respectively (all F<1.003, P>0.300) (Figures 2, 3).

The HOAD activity changed significantly with assay temperature only in the skeletal muscle of the two species ( *P* *. erythrurus* : F_1,18_=15.604, P=0.001; *P* *. przewalskii* : F_1,17_=5.622, P=0.033) (Figure 3), and not in liver ( *P* *. erythrurus* : F_1,18_=2.328, P=0.144; *P* *. przewalskii* : F_1,16_=0.394, P=0.539) (Figure 2). The HOAD activity of *P* *. erythrurus* in skeletal muscle was significantly higher than in *P* *. przewalskii* at 20 (F_1,16_=7.965, P=0.012) and 30° C (F_1,16_=7.642, P=0.014). However, there was no significant variation between livers of the two species at 20 or 30°C (all F<1.117, P>0.305). The thermal sensitivity of four enzyme activities, expressed as Q_10_ values calculated between 20 and 30°C, were compared between the two species for liver and skeletal muscle. Q_10_ values of each enzyme activities were similar between the two assay temperatures (all P>0.05) (Table 4).

### The expression of PGC-1α and PPAR-γ mRNA

**Figure 4:**
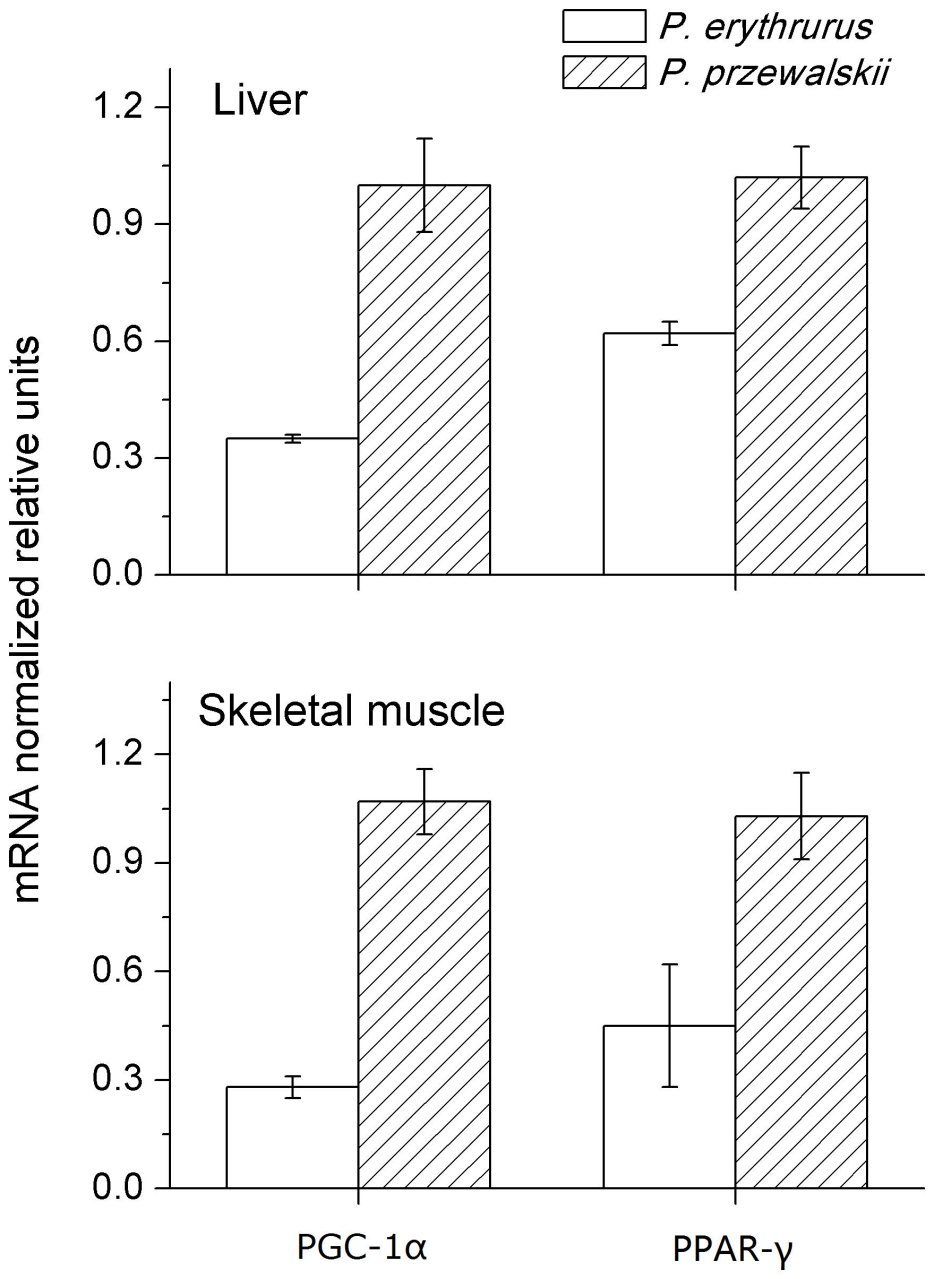
Real-time PCR analysis of PGC-1α and PPAR-γ mRNA levels in liver and skeletal muscle from *Phrynocephalus* *erythrurus* and *Phrynocephalus* *przewalskii* . Values are means ± s.e.m. in each group.

In liver, the expression of PGC-1α (F_1,12_=5.361, P<0.001) and PPAR-γ (F_1,12_=3.479, P<0.001) mRNAs was significantly higher in *P* *. przewalskii* , with 2.9-fold more PGC-1α and 1.6-fold more PPAR-γ in *P* *. przewalskii* compared to *P* *. erythrurus* , respectively (Figure 4). The expression of these two genes in skeletal muscle followed a similar pattern, with a lower expression level of the PGC-1α (F_1,11_=2.669, P<0.01) and PPAR-γ (F_1,11_=1.284, P<0.01) mRNAs in *P* *. erythrurus* (Figure 4).

## Discussion

To our knowledge, *P* *. erythrurus* dwells at altitudes higher than any other living lizards in the world. The present study is considered to be the first work which measures mitochondrial respiratory rate, metabolic enzymes and related genes simultaneously in a reptile species living at an altitude above 4000 m. Our results indicated that *P* *. erythrurus* has certain unique metabolic adaptive characteristics at the enzyme, mitochondrial and genetic levels.

### Low respiratory rate and high ATP production efficiency of mitochondria in *P* *. erythrurus*

The protein-specific maximal oxidative capacities of skeletal muscle mitochondria in *P* *. erythrurus* were only about 50% of the capacities of the mitochondria from *P* *. przewalskii* . Mitochondria from *P* *. erythrurus* liver present an even lower maximal respiratory rate, being 12~18% of those from the liver of *P* *. przewalskii* . The mitochondrial respiratory rate in *P* *. erythrurus* was also lower than some other reptiles in hibernation or cold seasons [34,35]. In general, the mitochondrial respiratory rate is obviously different among vertebrates, with the exact rate depending on the phylogeny and environmental conditions [10,36,37]. The mitochondrial respiratory rate of many ectothermic animals living in cold environments is lower than that of ectothermic animals living in warm environments [30]. Our results were consistent with these. Additionally, mitochondria are also believed to be an oxygen-sensitive element in cells, and mitochondrial respiratory rate and efficiency could decline at low PO_2_ [1]. Therefore, it is likely that the reduced respiratory rate of mitochondria in *P* *. erythrurus* could be a result of the synergistic effects of long-term living under cold temperature and low PO_2_ at high altitude. Furthermore, such variation at mitochondrial level may be directly related to the effect of temperature on mitochondrial membrane structure or the activity of the electron transport chain [34,38]. However, we do not know the structure of the mitochondrial membrane, and membrane potential can be affected by low temperature or low oxygen, thus causing depression of mitochondrial metabolism. These aspects will be the subject of particular attention in our future work.

Although the mitochondrial respiratory rate of *P* *. erythrurus* was much lower than many ectothermic animals, our results for proton leak indicated that liver mitochondrial efficiency was significantly higher in this species. The proton leak of liver mitochondria was estimated to account for 7~8% and 24~26% of state3 in *P* *. erythrurus* and *P* *. przewalskii* , respectively. Furthermore, the proton leak of *P* *. erythrurus* liver mitochondria accounted for a greater proportion of state4 than in *P* *. przewalskii* (74~79% VS. 43~48%). These results on one hand are associated with a higher respiratory control rate (RCR) in *P* *. erythrurus* than that in *P* *. przewalskii* ; on the other hand, may also directly relate to the biological function of proton leak. Numerous studies suggested that the main functions of proton leak are heat generation and reducing the production of ROS [39]. So a higher proton leak of *P* *. erythrurus* in the resting state (state4) should contribute to produce more heat, therefore benefitting *P* *. erythrurus* by maintaining a suitable core temperature to withstand the low cave temperature (measured by iButton, with an average temperature below 6° C, which close to its lethal temperature). However, a higher proton leak may accelerate the utilization of nutrient and speed up the depletion time of intrinsic body energy stores, a greater fat utilization mentioned below could be an effective strategy to compensate such high-energy consumption during resting state. Furthermore, a proton leak that is reduced to less than 10% during the active state (state3) of *P* *. erythrurus* will improve the utilization of oxygen and ATP production. On the other hand, the proton leak of the two lizards accounts for almost equal proportions in either state4 or state3 in skeletal muscle. In accordance with our results, similar results were also predominantly found in some assays on mammalian skeletal muscles [14,40]. The possible explanation could be related with the constant average value of mitochondrial ATP turnover during the rest-to-exercise transition, while the rate of proton leak across the mitochondrial inner membrane (which is controlled by ATP turnover) also remains constant [14].

### Low anaerobic metabolism and possibly greater fat utilization in *P* *. erythrurus*

It is assumed that animals exposed to a cold or hypoxic environment over an evolutionary time frame have, through natural selection, developed and maintained characters that are beneficial to maintaining ATP synthesis despite continually limited oxygen availability [41]. Numerous papers suggest that anaerobic metabolism is not extensively used for long time ATP supplement in terrestrial vertebrates. In our present study, anaerobic metabolism in the liver of *P* *. erythrurus* is significantly lower than that in *P* *. przewalskii* , which is implied by lower LDH activity as well as a lower LDH/CS ratio in liver. These results do not conform to the expectation that LDH activity would increase in cold temperatures, as well as low PO_2_ conditions (Pasteur Effect) [31,35]. However, for better living in cold and hypoxia conditions, endemic animals inhabiting high altitudes have revealed a number of strategies to adjust the anaerobic metabolism level, including isozyme spectrum and specific expression of LDH-M and LDH-H [42–44]. Furthermore, in accordance with our results, similar results were also predominantly found in some native animals at high altitude and hibernating animals [17,45]. Therefore, low LDH activity in *P* *. erythrurus* might the result of selectively evolved under the cold and hypoxia pressure of the Qinghai–Tibet Plateau. Such low anaerobic metabolism is also thought to be related to a more efficient coupling between ATP demand and ATP supply, allowing for a more effective integration between glycolysis and oxidative metabolism [45]. In contrast, there is no significant variation in LDH activities in the skeletal muscle of the two lizards, in accordance with the similar thermal sensitivities of LDH. Both *P* *. erythrurus* and *P* *. przewalskii* are mainly dependent on short burst locomotors in the wild. So the non-sustained contraction and relaxation of skeletal muscle in *P* *. erythrurus* can be achieved by anaerobic energy production, and a similar phenomenon has also been reported in some other species [41].

Fatty acid oxidation in reptiles is expected to be less important than in mammals, because HOAD activity and the HOAD/CS ratio is lower in reptilian species than in other vertebrates [46,47]. However, a higher free fat acid (FFA) (data not shown) and HOAD activity, accompanied by a higher HOAD/CS ratio, were found in *P* *. erythrurus* , suggesting a substantial proportion of the energy supply may derive from lipid oxidation in this species. The similar results were also detected in *Tupinambismerianae* and some other reptiles [37,48]. HOAD activity indicates the relative oxidation capacity of fatty acid, although, unlike CS, it does not give an indication of total aerobic capacity, and only measures the portion of fat oxidation. Increased fatty acid metabolism in liver acts to diminish the use of glycogen reserves, and leads to the accumulation of more glycogen in the *P* *. erythrurus* liver (proved by liver transmission electron microscopy, data not shown). In addition, the increase of capacity for fatty acid oxidation in skeletal muscle would limit the use of modest glycogen reserves, and this pattern, together with the constancy of CS in both liver and skeletal muscle examined, emphasises the nature of aerobic metabolism in *P* *. erythrurus* .

### Low expression of metabolism-related genes in *P* *. erythrurus*

Studies on mammals have indicated that both low temperature and hypoxia could induce PGC-1α and PPAR-γ expression in various tissues. However, *P* *. erythrurus* living in cold temperature and low PO_2_ conditions showed no increased expression of PGC-1α and PPAR-γ. Such downregulation of gene expression could be related to its effective adjustment at proton leak and enzyme level, and high body temperature and enough oxygen availability in its habitat could also eliminate the influence of cold and low PO_2_ on the expression of PGC-1α and PPAR-γ in *P* *. erythrurus* . Firstly, we found that *P* *. erythrurus* could achieve a body temperature of over 30° C during activity in their habitat, even when the ambient temperature was only about 10~15°C (unpublished data). Secondly, studies on Phrynocephalus lizards indicate that some physiological traits were significantly changed only when the PO_2_ was as low as 60 mmHg [19]. Previous studies on the oxygen-dependence of respiration also indicate that changing atmospheric PO_2_ mainly has a significant impact on the PO_2_ of alveolar air and arterial blood rather than on the PO_2_ of cell or mitochondria, while mitochondria can achieve a maximum respiratory rate even when PO_2_ is at a level of 15 mmHg [11]. Therefore, these characteristics, which are indicative of physiological, behavioural and structural aspects of *P* *. erythrurus* , provide a considerable contribution to living at cold temperatures and low PO_2_ environmental conditions, rather than metabolism-related genes. However, the function and mechanism of these genes in animals endemic to high altitudes remains unclear.

## Conclusion

As few reptiles are distribute at high altitude, and studies of metabolism characteristics of reptilian species living at high altitude remain limited and unclear. In the present study, some unique metabolism characteristics at mitochondrial, enzyme and gene levels were found in *P* *. erythrurus* , including an increased proton leak during state4 and a reduced proton leak at state3, low anaerobic metabolism, a possibly increased fat utilization and less reliance on metabolism-related genes. Together with these results, these strategies in *P* *. erythrurus* may effectively compensate for the negative influence of cold and low PO_2_. However, some outstanding questions remain about this species, and further in-depth studies on mitochondrial membrane composition and proton potential dynamics are needed in our future work.
